# Giant colonic diverticulum: radiographic and MDCT characteristics

**DOI:** 10.1007/s13244-015-0433-x

**Published:** 2015-09-19

**Authors:** Abdel-Rauf Zeina, Ahmad Mahamid, Alicia Nachtigal, Itamar Ashkenazi, Mika Shapira-Rootman

**Affiliations:** Department of Radiology, Hillel Yaffe Medical Center, Hadera, Israel; Division of Surgery, Hillel Yaffe Medical Center, Hadera, Israel; Faculty of Medicine, Technion- Israel Institute of Technology, Haifa, Israel

**Keywords:** Giant colonic diverticulum, Large colonic diverticulum, Sigmoid colon, Diverticulitis, Diverticulosis

## Abstract

Giant colonic diverticulum (GCD), defined as a diverticulum larger than 4 cm, is a rare entity that is generally a manifestation of colonic diverticular disease. Because of its rarity and its variable and non-specific presentation, the diagnosis of GCD depends mainly on imaging findings. Knowledge of the spectrum of radiographic and CT features of the GCD is important in making the correct diagnosis and potentially preventing complications. This review focuses on imaging findings characteristic of GCD as well as its complications and radiographic mimics.

*Teaching points*

• *Giant colonic diverticulum is a rare complication of diverticulosis*.

• *The most common symptom is abdominal pain presenting in approximately 70 % of patients*.

• *Diagnosis is based on imaging findings with plain abdominal radiographs and MDCT*.

• *Treatment consists of enbloc resection of the diverticulum and affected adjacent colon*.

## Introduction

Giant colonic diverticulum (GCD), defined as a diverticulum larger than 4 cm, is a rare entity that is generally a manifestation of colonic diverticular disease [1, 2]. In most reported cases of GCD, the diameter ranges between 4 and 9 cm, yet diverticula as large as 40 cm have been described [3, 4]. GCD mostly present at the anti-mesenteric side, and involve the sigmoid colon in about 90 % of cases [3, 5]. GCD equally affect both genders and usually present during the 7th and 8th decades of life [2, 5, 6].

Since the first description of giant diverticules by Bonvin and Bonte in 1946 [7], many names have been used to describe GCD, including giant sigmoid diverticulum, giant gas cyst, and giant colon cyst [2]. The literature concerning GCD has consisted mostly of case reports and series, the largest being 17 patients [2, 6, 8]. A recently published systematic review included a total of 166 cases of GCD, identified in 138 studies [5]. Knowledge of the spectrum of radiographic and CT features of the GCD is important in making the correct diagnosis and potentially preventing complications. In this article, we describe the pathophysiology, clinical presentation and imaging findings characteristic of GCD as well as its complications and radiographic mimics.

## Pathogenesis and histology

The pathogenesis of GCD is unclear. One of the theories that has been postulated relates to a ball-valve mechanism by which gas enters, but is unable to leave the diverticulum. The passage of air in one direction results in pressure elevation and differences in the colon, and subsequently in the GCD [9]. Gradual enlargement of the diverticulum leads to an intermittently palpable mass, also referred to as a phantom tumour. A potential role for gas-forming organisms has also been suggested [5].

**Fig. 1:**
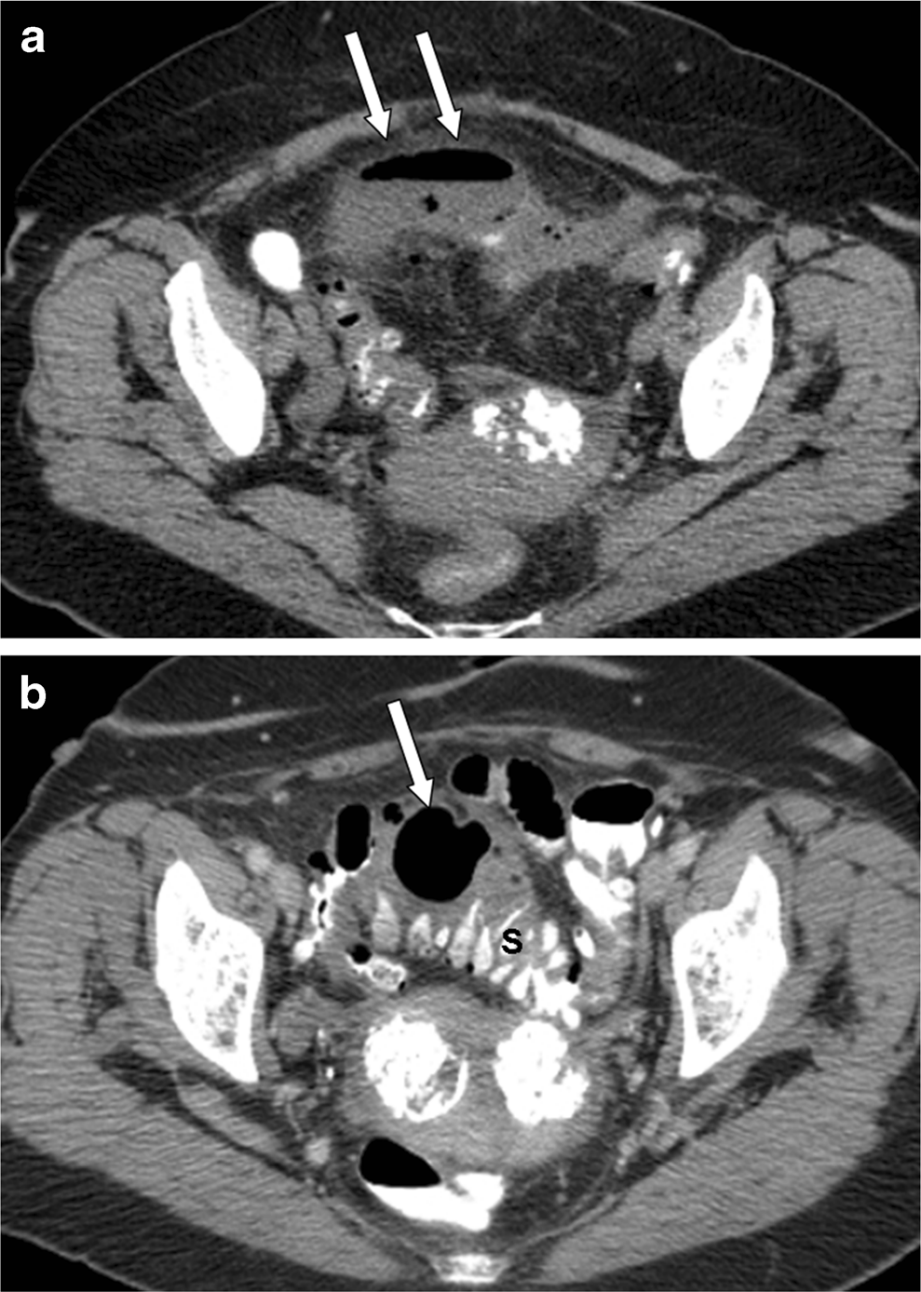
An 83-year-old woman with type II (inflammatory type) giant sigmoid diverticulum, which developed after an episode of acute diverticulitis. Axial (**a**) contrast-enhanced CT image of the abdomen shows a peridiverticular abscess (arrows), which was percutaneously drained. Axial (**b**) contrast-enhanced CT image obtained 5 months later (follow-up CT examination) shows a gas-filled structure adjacent to the sigmoid colon (arrow), without air–fluid level or pericolic inflammatory changes, consistent with type II (inflammatory type) GCD

A histological classification of GCD was first proposed by McNutt et al. [10] in 1988 and is still in use today. Three sub-types are described. Type 1 diverticula (22 %) represent a pulsion or pseudo-diverticula that contains remnants of muscularis mucosa and true muscularis. Type 2, which is the most common type (about two-thirds of cases [6]), is secondary to sub-serosal perforation, with subsequent formation of a walled-off abscess that gradually increases in size (Fig. 1). It contains only scar-tissue [3, 5]. Type 3 represents a true diverticulum that contains all four bowel layers and is most likely to have a congenital origin [3, 5]. It usually presents during childhood and accounts for 13 % of GCD [4, 11].

## Clinical presentation

Clinical presentation of GCD varies considerably: some patients are asymptomatic and GCD is discovered incidentally, some have chronic or sub-acute symptoms including non-specific abdominal complaints, and others present with fulminant and acute complications. Acute onset of abdominal pain affects approximately one-third of patients [5]. Complicated cases may present acutely with diverticulitis, perforation, ischemia, and bowel obstruction [1]. Chronic presentation affects approximately one-third of patients and is associated with intermittent abdominal discomfort, bloating, and constipation [5]. The most common clinical symptom is abdominal pain, which affects approximately 70 % of patients [5]. Additional symptoms include constipation, sensation of abdominal mass, vomiting, diarrhoea, and rectal bleeding [2, 5]. Other rare presentations reported in the literature include weight loss, urinary problems, intermittent abdominal mass accompanied by transitory leg swelling and focal neurological deficits, and intussusception [2, 12, 13]. Among the physical signs, the most common is abdominal mass followed by fever and tenderness [5]. A recent report documented nocturnal diarrhoea when sleeping on the right side as a symptom of GCD [14].

## Diagnostic modalities

Because of its rarity and its variable and non-specific presentation, the diagnosis of GCD depends mainly on imaging findings. Plain abdominal radiographs and multi-detector computer tomography (MDCT) are the main imaging modalities. Plain abdominal radiography remains a useful tool for first-line investigation due to its simplicity and widespread availability, while MDCT provides definitive diagnosis.

## Radiographic characteristics

On plain abdominal radiographs, GCD appears as a smooth-walled, gas-filled structure, round or oval, adjacent to the sigmoid colon (with or without air-fluid levels) [2, 3]. Also referred to as the "balloon sign" [15], this finding was evident in the plain abdominal X-rays of 99 % (103/104) of patients who underwent radiography and who were included in a review of case studies of GCD [6]. However, our more recent series of 17 patients revealed this sign in only 76 % (13/17) [2]. This suggests that before the widespread use of CT, radiographs apparently missed cases of GCD.

**Fig. 2:**
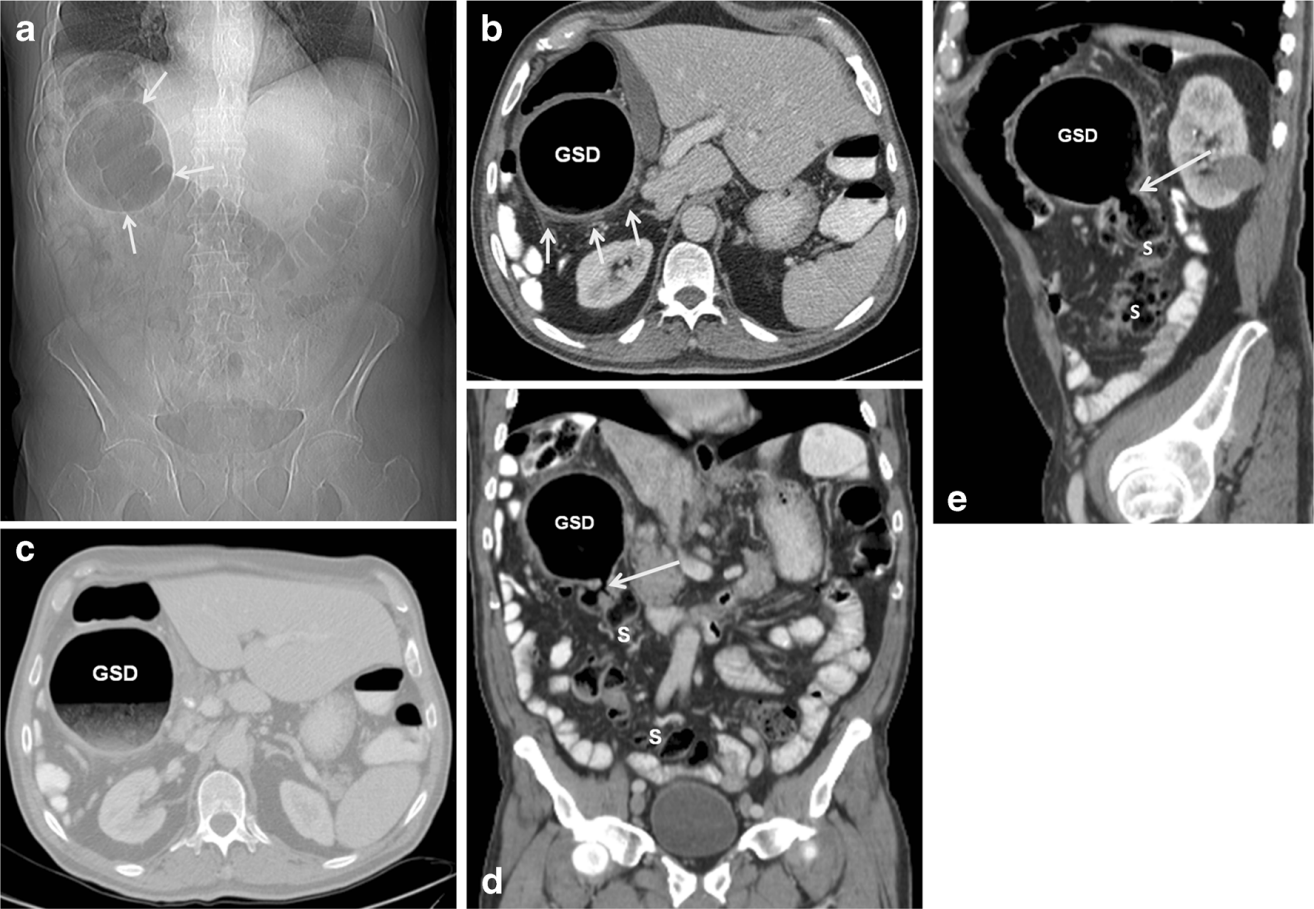
A 55-year-old man with giant sigmoid diverticulum (GSD) associated with acute diverticulitis. Abdominal radiograph (**a**) shows a large, round, homogenous radiolucency in the right upper quadrant that is smoothly marginated (arrows). Axial (**b** and **c**), coronal (**d**), and sagittal (**e**) contrast-enhanced CT images through the upper abdomen show a predominantly gas-filled structure in the right upper abdomen, communicating with the sigmoid colon (S) and consistent with GSD. The sigmoid colon is located in the right side of the abdomen (anatomical variant). The arrow demonstrates the neck of the GSD, which connects the diverticular cavity with the adjacent sigmoid colon; this finding is essential for correct diagnosis. The thickened wall of the diverticulum and the surrounding mesentery infiltration denote acute diverticulitis

**Fig. 3:**
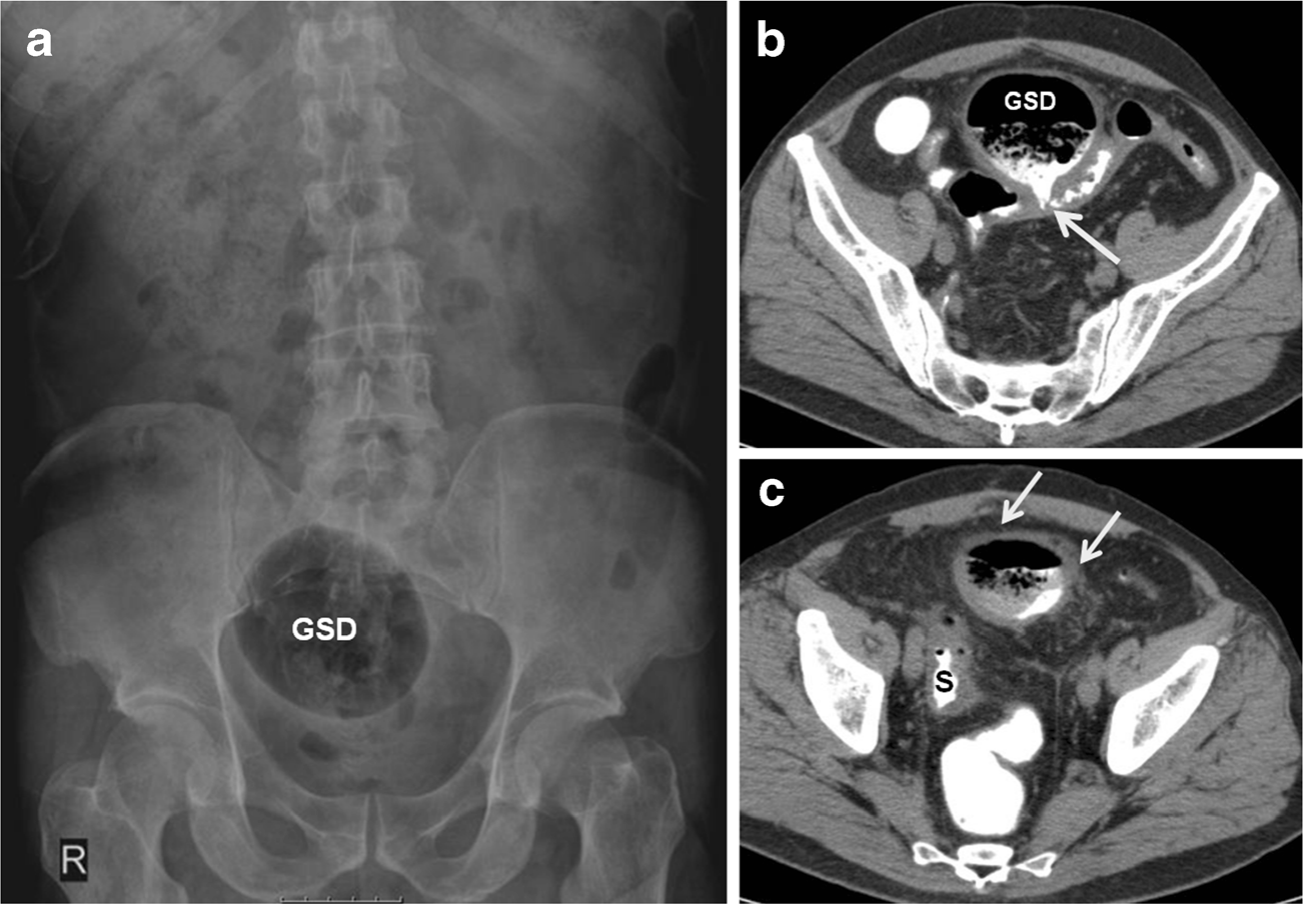
A 68-year-old man with giant sigmoid diverticulum (GSD) associated with acute diverticulitis. Abdominal radiograph (**a**) shows a large air-filled structure throughout the right pelvis, consistent with GSD. Axial (**b** and **c**) contrast-enhanced CT images through the pelvis show a predominantly gas-filled structure with air–contrast material level arising (arrow) from the sigmoid colon (S). The thickened wall of the diverticulum and the surrounding fat infiltration denote acute diverticulitis (arrows)

The larger the diameter of the GCD, the greater the chance that it will be detected on plain films [2] (Figs. 2 and 3). Lack of haustral folds and location in the lower abdomen may assist in differentiating between GCD and sigmoid or caecal volvulus [16]. Upright abdominal X-rays or lateral views can demonstrate fluid level, though these positions are generally not performed. Pneumoperitoneum and pneumomesenterium are indications of perforation of the gastrointestinal tract and appear in about 8 % of radiographs of GCD [6].

Barium or water-soluble contrast enema can help to identify GCD [17] and to determine the size and characteristic of the wall, and whether there is malignancy. This procedure shows communication between the GCD and the bowel in almost two-thirds of reported cases [6]. The wall of the GCD should be smooth and regular. The flow of contrast material flow into the diverticulum, together with irregular borders, should raise suspicion of chronic inflammatory or neoplastic changes [2]. Non-filling of the GCD by contrast material may result from narrow or inflamed ostium [16]. Barium enema, or sigmoidoscopy, if the sigmoid area is involved, can rule out carcinoma within or distal to a GCD. The risk of perforation of the GCD has led to a decline in the use of barium enema, in favour of diagnosis by computed tomography [5].

## MDCT characteristics

Multi-detector computed tomography (MDCT) is the preferred imaging technique for GCD, as it is for other complications of colonic diverticular disease, such as abscesses and fistula [18]. MDCT is a non-invasive means of evaluating the presence of GCD, its size, location and connection to the bowel, contents, and wall thickness; as well as the surrounding mesentery such as thickened surrounding fat indicative of recent inflammation [1], and accompanying complications. CT has demonstrated high sensitivity [6], and is more effective than barium enema in identifying communication between the GCD cavity and the gastrointestinal tract [6, 19], and of diagnosing alternative conditions, important for differential diagnosis.

**Fig. 4:**
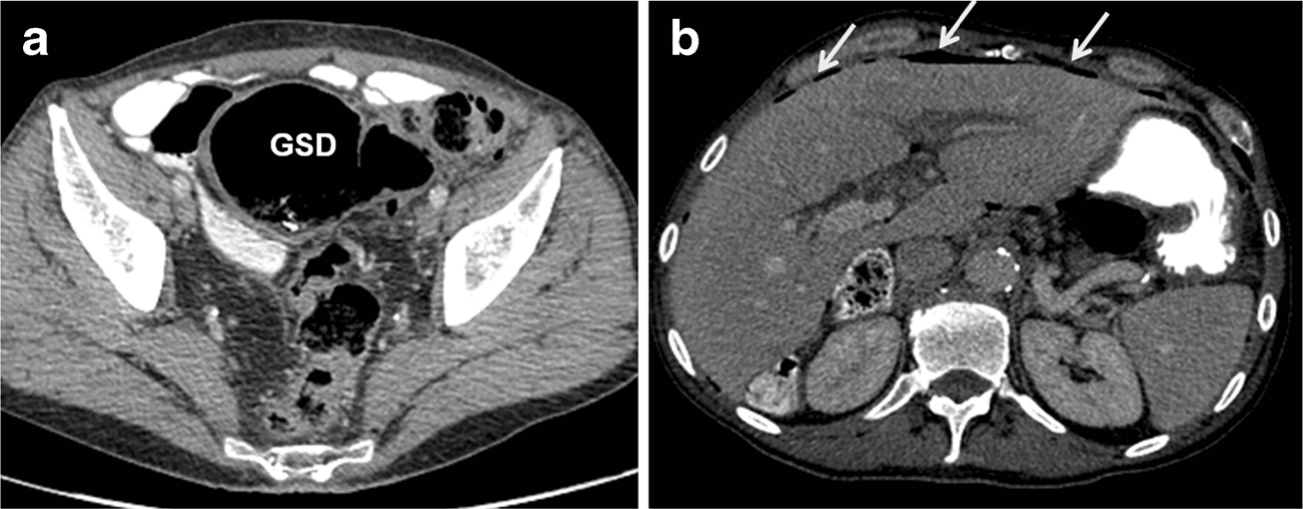
A 63-year-old man with giant sigmoid diverticulum (GSD) presented with acute abdomen. Axial contrast-enhanced CT images through the pelvis (**a**) and upper abdomen (**b**) show the GSD complicated with acute diverticulitis, perforation, and pneumoperitoneum (arrows)

On MDCT, the GCD usually appears as a predominantly gas-filled structure containing a small amount of fluid and communicating with the colon. Coronal and sagittal multiplanar reformatted (MPR) images are important for identifying the neck of the GCD, which connects the diverticular cavity with the adjacent colon; this finding is essential for correct diagnosis [2]. Administration of intravenous contrast material is helpful for differentiating between GCD and colonic perforation with abscess formation. Wall thickening and infiltration of the adjacent fat represent acute diverticulitis and localized peritonitis [2] (Figs. 2 and 4).

## Differential diagnosis and other diagnostic tools

Symptoms and signs of colonic diverticular disease and colorectal cancer are similar, and thus imaging is required to exclude malignancy. The general occurrence of GCD in the sigmoid colon may suggest diagnosis, but may also be misleading, since 10 % of GCD do not occur in the sigmoid colon.

**Fig. 5:**
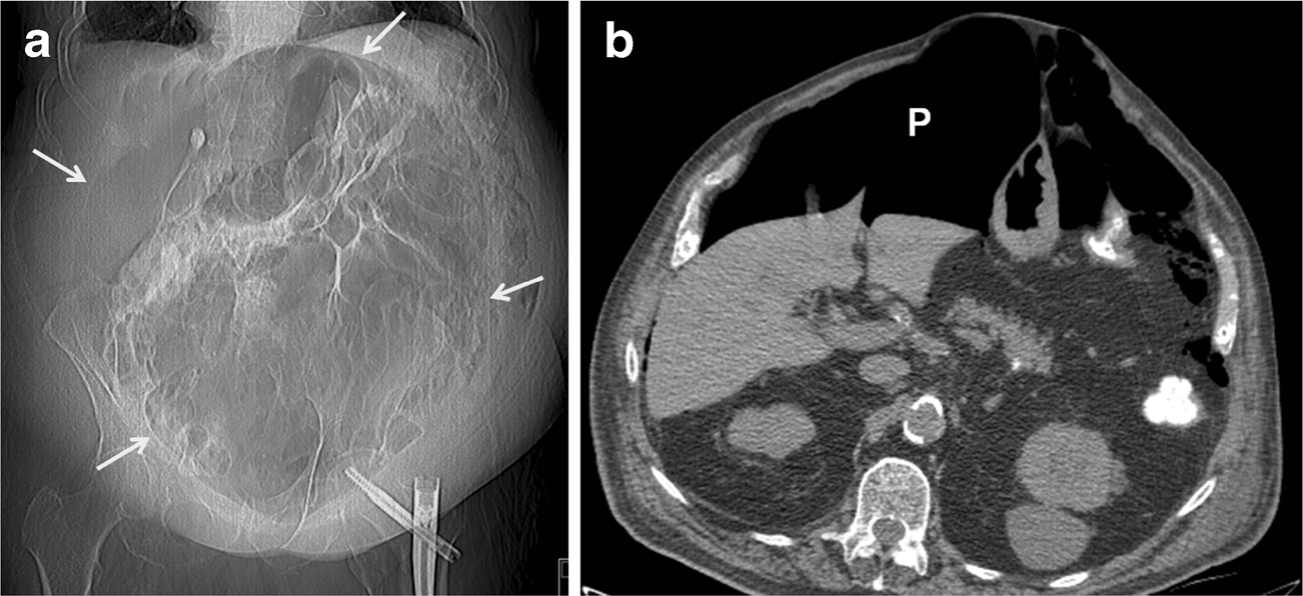
A 92-year-old man with massive pneumoperitoneum due to left colon perforation. CT surview of the abdomen (**a**) demonstrating the football sign (arrows) that may mimic giant colonic diverticulum. Axial unenhanced CT image through the upper abdomen (**b**) shows the massive pneumoperitoneum (P)

Radiological differential diagnosis should include abdominal abscess, localized pneumoperitoneum, large-bowel volvulus, enteric duplication cyst, Meckel's diverticulum, duodenal and jejunal diverticula, pneumatosis intestinalis, emphysematous cholecystitis, emphysematous cystitis, and infected pancreatic pseudocyst. However, on radiographs, duplication cysts are rarely found on the sigmoid colon and Meckel's diverticulum is generally located in the distal ileum and affects young children. CT scan of the abdomen may determine the differential diagnosis of GCD (Fig. 5). On CT, duplication cysts are usually fluid filled and fusiform, contrasting with round or oval gas-filled GCD. Further, the quantity of gas is generally greater in GCD than in abscesses; the explanation of such lies in the connection between the GCD and the colonic lumen.

Colonoscopy is a widely available and effective tool for assessing the colonic lumen and for ruling out carcinoma. However, it is not very useful for diagnosing GCD, since the connection point between the GCD and the colon cavity is small and hard to detect. In addition, colonoscopy is generally avoided due to the risk of perforation [5]. Further, the diverticular ostium is frequently small and easily missed [6, 13, 20].

## Complications

Perforation and abscess formation are the most common complications of GCD. Among the less frequent complications are peritonitis [2, 21], intestinal volvulus [22], intestinal obstruction [23, 24], lower gastrointestinal bleeding [25], and lymphoma or adenocarcinoma arising in the GDC [6, 21, 26]. We were able to find only one documented case of a patient with GCD and a concomitant metastatic rectal carcinoma [27]. The appearance on CT of irregular thickening of the upper rectal wall raised suspicion of malignancy, which was confirmed by fine needle aspirate from thorax nodules.

## Treatment

The overall approach to GCD depends on two aspects: symptoms and urgency. The recommended surgical management for symptomatic non-complicated GCD is elective primary resection of the diverticulum with the affected adjacent colon and primary anastomosis, with or without temporary protective ileostomy [24, 28–30]. In emergency cases of symptomatic complicated GCD, mainly due to perforation and secondary peritonitis, en bloc resection of the diverticulum and the affected colon and terminal temporal colostomy, with or without mucosal fistula, is the safest treatment [31]. Yet its disadvantage is the need for a second complex surgical procedure to restore intestinal continuity. For asymptomatic GCD, elective segmental colonic resection is still highly recommended. However, non-surgical conservative management should be considered for high-risk patients who are unable to tolerate surgery or who are unwilling to have surgery.

## Conclusion

Because of the rarity and differential symptomatology of giant colonic diverticulum, awareness of its imaging features is important. Plain abdominal radiographs may indicate giant colonic diverticulum; MDCT is the definitive imaging technique.
